# Infant feeding: emerging concepts to prevent HIV transmission

**DOI:** 10.1097/QCO.0000000000000986

**Published:** 2023-10-27

**Authors:** Alasdair Bamford, Caroline Foster, Hermione Lyall

**Affiliations:** a Great Ormond Street Hospital for Children NHS Foundation Trust; b UCL Great Ormond Street Institute of Child Health; c MRC Clinical Trials Unit at UCL; d Imperial College Healthcare NHS Trust, St Mary's Hospital, London, UK

**Keywords:** antiretroviral therapy, breastfeeding, HIV, postnatal prophylaxis, vertical transmission

## Abstract

**Purpose of review:**

HIV screening in pregnancy, universal suppressive antiretroviral therapy (ART) and breastfeeding avoidance can almost completely prevent vertical transmission of HIV. Breastfeeding is associated with an additional risk of transmission, although this risk is extremely low with suppressive maternal ART. This minimal risk must be balanced with the benefits of breastfeeding for women living with HIV (WLHIV) and their infants. Guidance in high-income countries has evolved, moving towards supported breast feeding for women on suppressive ART.

**Recent findings:**

Breastmilk transmission accounts for an increasing proportion of new infant infections globally. The majority of transmission data comes from studies including women not on suppressive ART. Breastmilk transmissions in the context of undetectable viral load have rarely occurred, although risk factors remain unclear. Outcome data on supported breastfeeding are accumulating, providing evidence for guidelines and informing infant feeding decisions. Long-acting ART for maternal preexposure prophylaxis or treatment, and infant postnatal prophylaxis are promising future options.

**Summary:**

Breastfeeding on suppressive ART has a very low risk of vertical transmission and can have multiple benefits for WLHIV and their infants. However, caution is advised with relaxation of breastfeeding guidance so as not to jeopardise the global goal of elimination of vertical transmission by 2030.

## INTRODUCTION

The WHO target for elimination of vertical transmission of HIV is less than 2% for nonbreastfeeding infants, and less than 5% for breastfed infants [1]. However, in many high-income countries (HIC), overall transmission rates are already less than 0.4% [2]. In the French cohort of over 5000 mother-infant pairs with maternal viral suppression on antiretroviral therapy (ART) in pregnancy, with infants formula fed, there were zero transmissions [3]. The efficacy and safety of modern ART, achieving maternal viral suppression in pregnancy and beyond, alongside progress towards normalization of pregnancy and delivery for women living with HIV (WLHIV) in HIC, has led to increasing demand from women and clinical teams to support breastfeeding in the context of viral suppression. However, there remains much to be elucidated regarding breastmilk transmission, including the impact of maternal and infant immunity/inflammation, viral compartmentalization and infant gut integrity.

We are a long way from achieving the global target of elimination of vertical transmission of HIV by 2030 [1,4]. To date, all clinical trials of mother/infant ART during breastfeeding have been in low and middle-income countries (L/MIC) demonstrating high efficacy, but not zero transmission. In many high prevalence L/MIC countries, the recommendation for universal breastfeeding is appropriate, irrespective of maternal ART status and viral load. In contrast, in HICs, the balance of minimal risk (breastfeeding on suppressive ART) versus no risk (formula feeding) requires additional consideration for families and clinical teams. Recent publications relevant to HIC and L/MIC are summarized below, and the range of current guidelines in different regions compared (Table 1) [5–8]. 

**Box 1:**
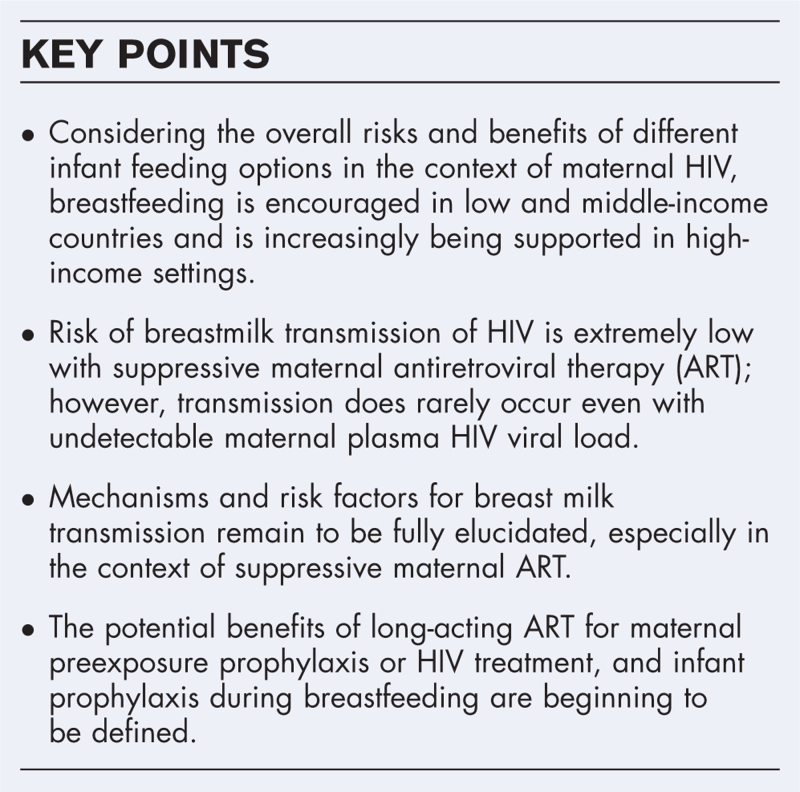
no caption available

**Table 1:** Summary of a range of current guidelines highlighting the differences between guidance for low and middle-income countries and high-income countries and variation in guidance between high-income countries

	WHO 2021 [5]	UK (BHIVA 2020) [6]	USA (DHSS 2023) [7]	Switzerland (FCSH 2019) [8]
General recommendation	Supported breastfeeding recommended for all	Formula feeding recommended Informed decision to breastfeed should be supported if virologically suppressed on ART with good maternal adherence	Formula or banked pasteurised donor human milk feeding recommended Informed decision to breastfeed should be supported for those with sustained undetectable VL	BF should not be actively recommended. Shared decision making resulting in a decision to BF should be supported.
Duration of BF	BF for at least 12 months or longer. Exclusive BF for 6 months, complementary foods from 6 months.	BF for as short a time as possible. Exclusive BF for first 6 months. Complementary foods from 6 months.	Exclusive BF for 6 months. Complementary foods from 6 months.	No specific guidance provided on duration or mixed feeding. 4 months exclusive BF mentioned for the general population.
Maternal ART recommendation	Universal urgent ART for all those breastfeeding with informed decision making about ART regimen choice	Universal ART for pregnant women for life. Choice of ART regimen individualised to patient taking into account concerns and preferences.	Universal ART for pregnant and breastfeeding women. Individualised choice of ART regimen using shared decision making.	Universal ART for pregnant and breastfeeding women. Standard ART regimen, preferentially with drugs with long-standing experience in pregnant women.
Antenatal monitoring	Maternal VL at/after 34 weeks gestation	Maternal VL 2–4 weeks after commencing ART (if starting in pregnancy) and at least once each trimester and at 36 weeks gestation	Maternal 2--4 weeks after initiating ART, monthly until undetectable, and at least every 3 months thereafter and at 36 weeks gestation or 4 weeks before delivery	Regular follow-up of treatment during pregnancy. At least two consecutive measurements before birth (minimal interval of 4 weeks and the last measurement after week 36 of pregnancy).
Standard PNP	Low risk: 6 weeks infant NVP High risk^a^: 6 weeks infant AZT/NVP followed by AZT/NVP or NVP alone for 6 weeks	Very low risk^b^: 2 weeks infant AZT Low risk^c^: 4 weeks infant AZT High risk: BF not recommended (4 weeks AZT/3TC with 2 weeks NVP)	Low risk^d^: 2 weeks AZT (consider extending to 4–6 weeks, or 6 weeks NVP when BF) Not low or high risk but maternal VL < 50 c/ml after 36 weeks gestation: 4–6 weeks AZT High risk: BF not recommended (presumptive treatment 3 drugs)	Optimal scenario^e^: no PNP Suboptimal scenario: BF not recommended. AZT/3TC/NVP or AZT/3TC/RAL. Discuss duration with specialist in paediatric infectious diseases.
Enhanced/extended PNP	Extending prophylaxis for the duration of BF can be considered	Not recommended to extend PNP beyond 4 weeks	Consider NVP for duration of BF	Not recommended
Day of delivery and postnatal maternal VL monitoring when BF	Maternal VL on day of delivery if not done in preceding 4 weeks. Repeat VL 3 months after delivery then every 6 months	Maternal VL on day of delivery then monthly for duration of BF and for 2 months after cessation	No specific mention of day of delivery maternal VL Maternal VL every 1–2 months during BF	No specific mention of day of delivery maternal VL Maternal VL Initially monthly then 2–3 monthly during BF
Maternal viraemia during breastfeeding	Consider reinitiating enhanced PNP if viraemia > 1000 copies/ml Continue BF with adherence support	Stop BF if detectable (no cut-off given) Offer cabergoline Consider infant postexposure prophylaxis	Stop BF (no cut-off given) Consider possibly restarting BF if maternal viraemia low level/transient	Stop BF if >50 copies/ml
Infant HIV testing if BF	NAT 0–2 days NAT 4–6 weeks NAT 9 months Ab 18 months (or 3 months after cessation of BF whichever is later)	PCR 0–2 days, 2 weeks, then monthly until 2 months after cessation of BF Ab at 2 years or minimum 2 months after cessation of BF whichever is later	Virological testing at birth, 14–21 days, 1–2 months and 4–6 months. Minimum every 3 months during BF. 4–6 weeks, 3 months and 6 months after cessation.	PCR birth from cord blood PCR 1 month 2 additional PCR e.g. 2 months and 4 months PCR 6 months Ab 18–24 months HIV test 3 months after weaning
Infant co-trimoxazole	Administer until exclusion of HIV infection after cessation of BF	Not recommended unless infant confirmed to have HIV infection	All high-risk infants at age 4 to 6 weeks, unless HIV has been fully excluded	No guidance given
Additional note		Additional recommendations for periods of mastitis or other intercurrent illness in mother or infant	Additional recommendations for periods of mastitis	Additional recommendations for periods of mastitis or infant hematemesis/melena

3TC, lamivudine; Ab, antibody; ART, antiretroviral therapy; AZT, zidovudine; BF, breastfeeding; BHIVA, British HIV Association; DHSS, Department of Health and Human Services; FCSH, Federal Commission for Sexual Health; HIC, high income country; L/MIC, low and middle-income country; NAT, nucleic acid amplification; NVP, nevirapine; PNP, postnatal prophylaxis; RAL, raltegravir; VL, viral load.

a Defined as: born to women with established HIV infection who have received less than 4 weeks of ART at the time of delivery; or born to women with established HIV infection with viral load >1000 copies/ml in the 4 weeks before delivery, if viral load is available; or born to women with incident HIV infection during pregnancy or breastfeeding; or born to women identified for the first time during the postpartum period, with or without a negative HIV test prenatally.

b Defined as: maternal VL not known to be or suspected to be >50 c/ml at delivery AND baby born after 34 weeks gestation AND maternal ART > 10 weeks duration AND two maternal VL results available <50 c/ml at least 4 weeks apart AND maternal VL <50 c/ml after 36 weeks gestation.

c Defined as: does not fulfil criteria for very low risk but most recent maternal VL < 50 c/ml OR low risk criteria met but delivery day maternal VL > 50 c/ml OR maternal delivery day VL suspected to be > 50 c/ml (high risk) but subsequently shown to be < 50 c/ml.

d Defined as: ≥37 weeks gestation, at least 10 weeks maternal ART, achieved and maintained vial suppression, maternal VL < 50 c/ml at or after 36 weeks gestation, not acute HIV infection in pregnancy, good adherence.

e Defined as regular follow-up of treatment during pregnancy (e.g. every 2–3 months) by a physician with expertise in the field of HIV is ascertained. HIV pVL is < 50 copies/ml ideally throughout pregnancy, but at least at the last two consecutive measurements before birth (minimal interval of 4 weeks and the last measurement after week 36 of pregnancy)

^∗^Note on terminology: This review primarily discusses and refers to pregnant and breastfeeding women throughout. The authors and editors fully acknowledge that the majority of the information presented will also apply to pregnant transgender men and gender-diverse individuals and their feeding choices, and that the terms chestfeeding and chestmilk are also used. We endorse the use of person-centred, nonstigmatising, gender-inclusive language in healthcare settings according to an individual's preference.

## MECHANISMS OF BREASTMILK TRANSMISSION OF HIV

There are multiple determinants of breastmilk transmission of HIV including virus-specific and maternal/infant factors. The highest transmission risk occurs with high maternal viraemia, commonly associated with primary infection. Breast tissue inflammation, including clinical/subclinical mastitis (SCM), also increases transmission risk. Breastmilk transmission may be due to cell-associated or cell-free virus and integrity of infant gut mucosa is an important factor; infections and/or foods, which compromise this may promote transmission [9,10].

The contribution of HIV drug resistance mutations (DRMs) to breastmilk transmission is unclear. However, a case-controlled substudy of the Promoting Maternal and Infant Survival Everywhere (PROMISE) trial (*n* = 37), which involved sequential randomizations of mother/infant pairs antepartum: to one of three maternal treatment regimens (zidovudine with single-dose nevirapine at the onset of labour and tenofovir/emtricitabine tail versus zidovudine/lamivudine/lopinavir-ritonavir versus tenofovir/emtricitabine/lopinavir-ritonavir) and postpartum: to maternal tenofovir/emtricitabine/lopinavir-ritonavir versus infant nevirapine, for the duration of breastfeeding, demonstrated maternal DRMs [predominately nonnucleoside reverse transcriptase inhibitor (NNRTI)] to be an independent risk factor. Infected infants had significant rates of NNRTI resistance, compromising future ART options. This also has implications for postnatal prophylaxis (PNP) agent choice in the context of documented maternal genotypic resistance. This study reconfirmed higher maternal viraemia during breastfeeding to be associated with increased transmission risk [11^▪▪^].

Maternal HIV and/or ART (or other antimicrobials) may affect breastmilk composition and the infant microbiome [12,13] with potential implications for infant health, growth and development. How this impacts HIV transmission risk remains to be determined.

The pharmacokinetics/pharmacodynamics of ART in breastmilk has important implications for transmission, as well as resistance and drug toxicity in breastfeeding infants. A study of 21 mother/infant pairs demonstrated that maternal plasma/breastmilk ratios vary between agents, breastmilk transfer being significant for rilpivirine, efavirenz, nevirapine, abacavir, lamivudine, emtricitabine, tenofovir alafenamide and raltegravir but minimal for tenofovir disoproxil, dolutegravir, bictegravir and darunavir/ritonavir. Agents were detectable in infant plasma, below the exposure index of 10% (safety threshold for infant exposure to maternal drugs through breastmilk). Infant plasma concentrations did not necessarily correlate with those in breastmilk [14^▪^].

Maternal/infant immunity may also play a key role. A substudy of the Breastfeeding, Antiretroviral, and Nutrition Study (BAN) found, in the absence of maternal ART, a combination of exposure to maternal HIV strains more susceptible to antibody-dependant cytotoxicity (ADCC) combined with higher infant breadth and potency of ADCC was associated with lower breastmilk transmission [15]. Additional analysis from BAN and the Center for HIV/AIDS Vaccine Immunology 009 (CHAVI009) cohorts has investigated the role of maternal plasma broadly neutralizing HIV antibodies (bNAb), their impact on viral selection and breastmilk transmission. Results indicated that both multispecific broad activity and uncommon epitope-specific plasma bNAbs may protect against transmission, providing further evidence to direct potential bNAb strategies for prevention of viral transmission [16].

Subclinical mastitis may increase breastmilk transmission. A sub-study of ANRS12174 (270 mothers not on ART) measured breastmilk sodium and potassium (weeks 14, 26 and 38 postpartum) to determine SCM incidence and effects on HIV shedding. Recurrent severe SCM significantly increased breastmilk inflammatory cytokines, cell-free and cell associated HIV [odds ratio (OR) 5.2; 95% confidence interval (95% CI): 1.7–15.6]. HIV shedding increased seven-fold from mild to severe SCM: median 41 copies/ml (c/ml) (IQR: 0–179) versus 309 c/ml (IQR: 18–1940) compared to 0 c/ml (IQR: 0–71) in women without SCM. All infants received PNP, and transmission was low (1.5%); therefore, it was not possible to link SCM with transmission risk [17^▪▪^].

**FIGURE 1:**
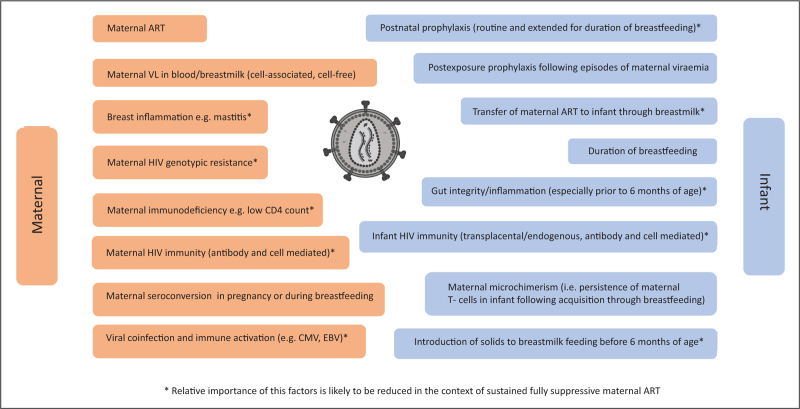
Main factors potentially influencing breastmilk transmission of HIV. ART, antiretroviral therapy; CMV, cytomegalovirus; EBV, Epstein--Barr virus; VL viral load.

Maternal viraemia is the dominant determinant of breastmilk transmission, but further studies of maternal viral characteristics, pharmacokinetics/pharmacodynamics and maternal/infant immune and inflammatory responses will inform future strategies to minimise breastmilk transmission. The main factors potentially influencing breastmilk transmission of HIV are summarised in Fig. 1.

## CLINICAL STUDIES IN LOW AND MIDDLE-INCOME COUNTRIES

Incident maternal HIV infection during breastfeeding now accounts for a substantial proportion of new infant infections [18]. WHO guidelines recommend retesting for postpartum women in high-HIV-burden settings [5] and this is now implemented in many countries [19–21]. Point-of-care (POC) HIV viral load testing at delivery and subsequent routine care visits is in trial to promote maternal adherence, facilitate enhanced PNP for breastfed infants and rapid ART initiation following infection [22–24].

Clinical trials of maternal preexposure prophylaxis (PrEP) during pregnancy and breastfeeding are being prioritized, and to date demonstrate no significant safety concerns. Suboptimal adherence limits efficacy of oral and vaginal PrEP, highlighting potential benefits to long acting injectable (LAI) PrEP (e.g. cabotegravir). Clinical trials linking LAI PrEP with routine healthcare visits to reduce new maternal infections during breastfeeding are underway [25].

In the context of an undetectable maternal viral load, breastmilk transmission is rare, but has occurred. In the postnatal part of the PROMISE trial (NCT01061151), 2431 breastfeeding mother/infant pairs were randomized to maternal ART or infant PNP; both were equally efficacious with an overall transmission rate of 0.6% (95% CI: 0.3–1.3) at 12 months. Overall, 14 infants were infected, seven in each arm. With maternal ART, postnatal infection was detected in two infants when maternal viral loads were less than 40 c/ml (at 13 and 38 weeks postpartum) [26,27]. Dolutegravir in Pregnant HIV Mothers and Their Neonates-2 (DolPHIN-2) (NCT03249181) randomized 268 women in the third trimester, to dolutegravir or efavirenz-based ART. Three infants were infected *in utero* in the dolutegravir arm. In the efavirenz arm, one breastfed infant was diagnosed at 72 weeks; all maternal viral loads (birth, 12, 24, 48, 72 weeks) were less than 50 c/ml, and infant VLs at birth, 6, 12, 24 and 48 weeks were negative. The infant was exclusively breastfed until 24 weeks, followed by mixed feeding, and there was no clinical mastitis, surrogate breast-feeding, nor other known exposures. Infant and maternal virus had the same genetic sequence [28^▪^].

The factors which drive rare breastmilk transmissions despite sustained maternal viral suppression may include intermittent maternal adherence with undetected viral rebounds, SCM, discordant plasma/breastmilk viral load, cell-associated transmission, altered infant gut integrity, or indeed ART in pregnancy and breastfeeding may supress infant viraemia following in-utero transmission.

## POLICIES AND CLINICAL STUDIES IN HIGH-INCOME COUNTRIES

With access to clean water and formula milk, HICs have historically recommended formula feeding for infants born to WLHIV. Recently, guidance has changed to a more nuanced approach, following risk stratification with women on suppressive ART being supported to breastfeed [6–8,29,30]. However, gaps in knowledge of women's experiences, beliefs and desires and effective risk-counselling around infant feeding remain [31].

Ongoing enhanced surveillance in the United Kingdom (UK) since 2012 recorded 111 breastfed infants, approximately 1.3% of infants born to WLHIV, with no HIV transmissions. Attendance issues were reported in one-third (25/77) of those undergoing monthly viral load testing. Breastfeeding ceased following maternal viral load rebound in 10 cases [32].

Postnatal prophylaxis guidance for breastfeeding infants in HIC varies widely from no PNP (Switzerland) [8], to 2 weeks zidovudine (UK) [6], to three drugs (zidovudine, lamivudine and nevirapine) for 4–6 weeks initially followed by nevirapine monotherapy continued through 6 weeks after discontinuation of breastfeeding (Baltimore, USA) [33]. Additionally, there is little consensus on frequency of maternal viral load monitoring and infant testing or guidance on starting infant postexposure prophylaxis (PEP) if maternal viraemia occurs during breastfeeding [6–8,29]. Monthly viral load was recommended in one U.S. cohort (*n* = 10) [33], contrasting with fortnightly maternal viral load and infant monitoring at months 1, 2, 4 then 3 monthly in another (*n* = 6) [34]. Between 2019 and 2021, 25 out of 41 (61%) Swiss WLHIV chose to breastfeed [median 6.3 months (IQR 2.5–11.1)] citing bonding and maternal/infant health as motivational factors. Additional formula milk was acceptable with no solids before 6 months. Two women developed viraemia; viral load 183 c/ml at 2 months with immediate cessation of breastfeeding, and viral load 63 and 79 c/ml at 5 and 8 months returning to less than 50 c/ml within 2 weeks. There were no transmissions [35]. Maternal viraemia (310–760 c/ml) (while on ART) during breastfeeding in a small European case series after routine infant PNP completion resulted in immediate cessation of breastfeeding and 4 weeks infant PEP for two infants. A third child aged 3 years received PEP following a new maternal HIV diagnosis (viral load 123 381 c/ml) during breastfeeding [36]. No transmissions occurred, but the latter case illustrates the need for PrEP access for pregnant and breastfeeding women at risk of acquiring HIV in all settings [25,37]. A recent systematic review highlighted the lack of data available to inform PrEP use for women in Europe during pregnancy and postpartum [38]. The inclusion in trials of the increasing number of women conceiving on LAI treatment or PrEP, alongside pharmacokinetic data during pregnancy and lactation is crucial [39].

## ONGOING TRIALS AND RESEARCH

The majority of recent/ongoing trials in L/MICs focus on behavioural interventions to improve maternal ART adherence and retention in care during breastfeeding or explore optimisation of infant PNP (Table 2). Studies in HIC focus more on ART pharmacokinetics, qualitative data and outcomes of observational cohorts, and low HIV prevalence and very low rates of breastmilk transmission on ART preclude randomized controlled trials. Data extrapolated from LMIC may overestimate risk in HIC due to variation in additional contributory factors such as infant gastrointestinal infection.

**Table 2:** Summary of relevant ongoing studies directly relating to prevention of vertical HIV transmission through infant feeding (Clinicaltrials.gov) Search strategy; clincialtrials.gov: HIV+ Breast feeding, HIV+ infant Infection; HIV +prevention infant infection; HIV+ pregnancy

Study title	NCT Number	Intervention and setting
Mobile phone text messaging plus motivational interviewing: Effects on breastfeeding, child health and survival outcomes, a group sequential randomised standard of care-controlled trial	NCT05063240	Behavioral Intervention: Mobile phone text messaging plus prospective motivational interviewing versus standard infant feeding counselling. South Africa, *n* = 275.
Understanding the Role of Food Insecurity and Depression in Nonadherence to Option B+ Among Perinatal Kenyan Women Living With HIV: A Syndemics Approach	NCT05219552	Behavioural Intervention: personalized lactation support and monthly unconditional cash transfers (100 USD/month) from approximately 30 weeks pregnancy to approximately 6-month postpartum versus current standard care. Kenya, *n* = 40.
Pharmacokinetic Properties of Antiretroviral and Anti-Tuberculosis Drugs During Pregnancy and Postpartum. IMPAACT 2026	NCT04518228	Observational: Phase IV observational study of antiretroviral therapy, including bictegravir, tenofovir alafenamide and cabotegravir. Maternal PK in pregnancy, postpartum and breast milk. Infant washout PK at birth and 5–9 days. USA, South America, Thailand, India, sub-Saharan Africa, *n* = 325
Therapeutic drug monitoring to optimize antiretroviral regimens in HIV-infected women who want to breastfeed. PANNA-B TDM	NCT05642481	Observational: Therapeutic drug monitoring in plasma of mother and child and in breastmilk. Netherlands, *n* = 32
Elimination of Paediatric HIV-1 Infection: Evaluation of the Prevention Programme and Rescue Intervention Based on the Expanded Programme on Immunization (EPI). ANRS 12388 PREVENIR-PEV Study.	NCT03869944	Phase 2b interventional study of maternal/infant HIV testing at 2-month EPI visit. Component 2 breast feeding mothers aim to reduce HIV-1 transmission to less than 3% between 2 and 12 months among exposed children who completed the second EPI visit. Infant PrEP (lamivudine) until 12 months of age (or end of breastfeeding) for women with unsuppressed HIV-1 infection (≥1000 copies of HIV-1 RNA/ml) with a child whose tests to date (EPI-2 visit) do not reveal the existence of infection. Burkina Faso *n* = 97
Piloting Risk Stratification and Tailored Interventions with pregnant and postpartum women with HIV in Kenya to prevent disengagement from care and viral failure	NCT05841797	Behavioural: pilot hybrid type 2 effectiveness-implementation trial in which pregnant women at higher risk for missed visits and treatment failure are randomized 1 : 1:1 to standard of care, in-person programme management+ or mobile programme management + and followed for 6 months postpartum. Kenya *n* = 120
Adherence to HIV Treatment Postpartum: The Implications of Transitions among women living with HIV in South Africa	NCT04846569	Behavioural: theoretically driven curriculum focused on supporting mothers from pregnancy through postpartum to promote sustained HIV treatment adherence versus enhanced standard of care. Outcome measures; acceptability, self-reported adherence, engagement in care and viral load to 6 months postpartum. South Africa n = 63
Evaluating the HITSystem to Improve PMTCT Retention and Maternal Viral Suppression in Kenya	NCT04571684	Interventional: cluster randomized controlled trial of eHealth intervention of SMS texts to patients and algorithm-driven electronic alerts for providers to increase retention in guideline-adherent prevention of mother-to-child transmission and to increase viral suppression and appropriate clinical action through 6 months postpartum, compared to standard of care. Kenya *n* = 1512
Implementing a Risk Score to Facilitate Enhanced Adherence Support for Pregnant and Postpartum Women at Risk of Viremia	NCT05845619	Behavioural: pilot study of enhanced virologic monitoring with peer counselling about viral load levels and rapid delivery of viral load results for pregnant women in third trimester or within 6 months of delivery. Primary outcome viral suppression 6 months following the intervention, compared to historical controls. Kenya *n* = 550

Passive infant immunisation with bNAbs has prevented transmission in nonhuman primates, including during breastfeeding [40] and early human safety and pharmacokinetic data is encouraging [41]. The bNAb VRC01 (subcutaneous day 5 then monthly) and its long-acting formulation VRC01LS (subcutaneous day 5 then 12 weekly) given to breastfed infants was well tolerated with protective levels persisting for 8 weeks in the majority [42,43]. VRC07–523LS, maintained levels out to 12 weeks suggesting 3-monthly dosing maybe feasible [44]. Modelling suggests bNAbs may be cost-effective for high-risk infants (efficacy >30%, cost <$200/dose) [45]. Practicalities including production and cold chain remain challenging. A Phase 1/2 safety/pharmacokinetics study of combination bNAbs (subcutaneous VRC07–523LS + CAP256V2LS) is enrolling and further single bNAb studies of subcutaneous VRC07–523LS and subcutaneous VRC01LS planned [46–48].

## ETHICAL AND LEGAL CONSIDERATIONS: OPTIMIZING HEALTH OUTCOMES WHILE BALANCING THE RIGHTS OF PARENTS AND CHILDREN

There remains a dilemma in the absence of clinical trials in HIC; what is an acceptable risk of breastfeeding transmission and how are the rights of parents balanced with that of the child? Formula feeding has zero transmission risk, but a significant financial and emotional cost. Health benefits of breastfeeding include reduced maternal cancer, diabetes and obesity, and reduced infections and improved intelligence quotient (IQ)/adult earning capacity for infants [49,50]. Guidelines recommend provision of free formula milk, but this is inconsistent, despite many families living in poverty [6,51].

In a UK survey of WLHIV, two-thirds reported being asked why they were not breastfeeding by community members, and one-third cited secrecy and HIV-stigma as reasons for wanting to breastfeed [52]. Women may choose to breastfeed to avoid potential disclosure of their HIV status to partners, family and society. This pressure continues whilst HIV remains a stigmatised condition. Persons with HIV from the USA had relatively favourable attitudes to breastfeeding, although one in 10 reported feeling coerced to formula feed [53]. Similar themes have been reported by women living in all settings [54]. A difficult ethical and clinical equipoise remains. Consider the rights of a father requesting a zero-risk strategy versus a mother who wants to breastfeed on suppressive ART, or conversely a woman very anxious regarding the risk of transmission but feels pressured to breastfeed by others. Qualitative studies hand-in-hand with stakeholder engagement are required to better understand how families living with HIV navigate this complex risk balance [55,56]. Of note, the voice of the infant at risk of HIV infection through breastfeeding is absent.

Supporting infant feeding choices requires a multidisciplinary approach respecting the autonomy of the individual within a framework of shared decision making and harm reduction [57]. As healthcare professionals, we must manage our own anxiety relating to the very low risk of transmission on suppressive ART versus zero risk of formula feeding.

## FUTURE DIRECTIONS

Although there are differences in resources, guidance and practice between HIC and L/MIC, research findings from all settings are relevant when providing parental counselling. With global ART rollout, breastmilk transmission of HIV for women already diagnosed will be an increasingly rare event. With improved access to low-cost effective ART, laboratory monitoring and well tolerated breastmilk alternatives, guidance may eventually converge across HIC and L/MIC [58].

In the absence of clinical trials, collection of large-scale observational data on feeding practices and transmission for mother/infant pairs will be essential to monitor the safety of breastfeeding. Single country case series in HIC are too small to draw robust conclusions on risk [33–35,59,60]. A large observational study is planned within the European Pregnancy and Paediatric Infections Cohort Collaboration (EPPICC). Long-term mother/infant health outcome data with suitable control groups will also help address the current gaps in knowledge regarding the risks and benefits of breastfeeding and to enhance informed decision making.

Adherence to ART for both mother and infant is challenging, and while studies of nonpharmacological measures are essential, long-acting agents including bNAbs, cabotegravir and lenacapavir have the potential to significantly improve outcomes [61]. Although challenging, studies of pharmacokinetics and safety of LAI agents in infants are eagerly awaited [48]. An effective vaccine against HIV infection remains elusive, but when available, vaccination to prevent maternal and/or infant HIV infection during breastfeeding should be explored as a priority.

Keeping families at the centre of efforts to develop newer strategies is key to success and they should be consulted at the earliest stages of study design. With relatively high rates of new maternal HIV acquisition during breastfeeding, access to PrEP services and clinical trials for women during this highly vulnerable period are essential [25,39]. Incident pregnancy outcome data including long-term infant HIV status, postnatal maternal, breast milk and infant pharmacokinetics should be planned for treatment studies enrolling participants with childbearing potential.

Further development of cost-effective, accessible POC viral load monitoring during breastfeeding will address some of the barriers to well tolerated feeding. This, alongside harmonised, evidence-based guidance on appropriate interventions at times of maternal viraemia, for example infant PrEP/PNP/PEP will further reduce the very low persisting risk of transmission.

## CONCLUSION

Although breastmilk transmission in the context of maternal ART is increasingly rare, transmission does still occur even when maternal plasma HIV appears fully suppressed. Elimination of breastmilk transmission of HIV will only be achievable with universal access to:

(1) ART for breastfeeding WLHIV
(2) effective PNP for infants at risk of transmission
(3) effective PrEP, HIV prevention services and regular screening for women at risk of incident HIV infection during breastfeeding
(4) well tolerated alternatives to breastfeeding when required

Policies and practice relating to infant feeding are evolving rapidly with the common aim of achieving zero vertical HIV transmissions while optimising long-term maternal/infant health and balancing the rights of both parents and children. As transmission events become increasingly rare, agile policies that respond to robust observational data will help ensure these goals are delivered.

## Acknowledgements

*None.*

### Financial support and sponsorship

*None.*

### Conflicts of interest

*There are no conflicts of interest.*

## REFERENCES AND RECOMMENDED READING

Papers of particular interest, published within the annual period of review, have been highlighted as:

▪ of special interest
▪▪ of outstanding interest
